# Post-COVID recovery: characteristics of chronically critically ill patients admitted to a long-term acute care hospital

**DOI:** 10.12688/f1000research.26989.2

**Published:** 2021-04-20

**Authors:** Meg Stearn Hassenpflug, Dale Jun, David R. Nelson, Tamas Dolinay

**Affiliations:** 1 Barlow Respiratory Hospital, Los Angeles, California, 90026, USA; 2 UCLA David Geffen School of Medicine, Los Angeles, California, 90095, USA

**Keywords:** COVID-19, post-acute, chronic critical illness, long-term acute care, mechanical ventilation, tracheostomy, recovery

## Abstract

**Background:** Survivors of COVID-19 pneumonia often suffer from chronic critical illness (CCI) and require long-term hospitalization. Long-term acute care (LTAC) hospitals are vital in the care of CCI patients, but their role for patients post COVID-19 infection is not known. Barlow Respiratory Hospital (BRH) is a 105-bed, LTAC hospital network serving ventilator-dependent and medically-complex patients transferred from the ICUs of hospitals in southern California. We report patient characteristics of our first series of COVID-19 survivors admitted to the post-acute venue of an LTAC hospital.

**Methods:** Single-center observational descriptive report of patients recovering from acute infectious complications of COVID-19 pneumonia requiring long-term respiratory support.

**Results:** From 28 April to 7 September 2020, 41 patients were admitted to BRH for continued recovery from COVID-19 pneumonia. Median age: 68 [44-94] years, 25/41 (61%) male, 33/41 (80.5%) with tracheostomy, 21/41 (51.2%) on invasive mechanical ventilation, 9/41 (22%) receiving hemodialysis. All mechanical ventilation and hemodialysis interventions were initiated at the transferring hospital.

**Conclusions:** To our knowledge, this is the first report to characterize CCI and medically complex COVID-19 patients transferred to the post-acute venue of an LTAC hospital. Patients on average spent over six weeks in the transferring hospital mostly in the ICU, are largely elderly, carry the known risk factors for COVID-19 infection, and experienced respiratory failure necessitating prolonged mechanical ventilation via tracheostomy. Our findings suggest that these patients will continue to require considerable medical interventions and treatments, including weaning from mechanical ventilation, owing to the numerous sequelae of the infection and the burden of acute-on-chronic diseases. As ICU survival rates improve, this research further emphasizes the important role of the LTAC hospital in responding to the COVID-19 crisis.

## Introduction

Advances in technology, research, and adoption of evidence-based practices have significantly improved intensive care unit (ICU) survivorship, creating the population of patients recognized as chronically critically ill (CCI) ^1^. This improved survival, however, is often accompanied by a prolonged and challenging course of recovery. This population now includes ICU survivors of coronavirus disease 2019 (COVID-19) in need of post-acute care for continued recovery from their infection. Long-term acute care (LTAC) hospitals are vital in the care of CCI patients ^2^, but their role for patients post COVID-19 infection is not known. Barlow Respiratory Hospital (BRH) is a 105-bed, not for profit, LTAC hospital network serving ventilator-dependent and medically complex patients transferred from the ICUs of hospitals in southern California. Herein, we report patient characteristics of our first series of COVID-19 survivors admitted to the post-acute venue of an LTAC, as an essential step in the continuum of care for treatment, rehabilitation, and recovery.

## Methods

### Study background

This is a single-center observational descriptive report of patients recovering from acute infectious complications of COVID-19 pneumonia requiring long-term respiratory support. Over half were admitted on invasive mechanical ventilation having experienced respiratory failure at the transferring hospital. Patients were admitted for attempts at weaning from prolonged mechanical ventilation, as well as for continued care and treatment of infections, complications, and co-morbid conditions.

### Ethical considerations

The study was approved by the Western Institutional Review Board (WIRB), reference: #1-1348082-1. Only de-identified health information was collected and recorded in the database to ensure patient privacy and data safety. The WIRB waived the need for consent from patients who participated in the study.

### Data collection and statistical analysis

Patients with at least one positive COVID-19 polymerase chain reaction testing (PCR) prior to admission to BRH were enrolled in the study on an ongoing basis. The tests were performed from nasopharyngeal, oropharyngeal or lower airway sampling. Exclusion criteria of the study was the absence of positive COVID-19 PCR testing prior to admission. This approach was followed to minimize biases in data collection. Due to the inherent false negative rate of the PCR testing, it is possible that we did not capture all previously COVID-19 positive patients ^3^. Patients were determined to be in the post-infective phase prior to transfer to BRH.

The data are reported with binary values. The 0 represents absence and 1 represents presence of a condition (see *Underlying data*). Missing data are reported as unknown. Data were collected from our electronic medical record system using a combination of automated data extraction and manual collection. We collected baseline demographics (age, gender, race/ethnicity, premorbid location), presence of known COVID-19 risk factors, events at transferring hospital, and descriptors of status on admission to the LTAC to construct the Barlow COVID-19 data set.

All statistical analysis was performed using Microsoft Excel 2013 program (Microsoft Corporation, Santa Rosa, CA). We used descriptive statistics to describe the basic features of the data. Missing data were omitted from analysis. For serum albumin and serum glucose, only n=36 and n=40 values were available respectively. No statistical comparisons were made. 

We used the STROBE cross sectional reporting guidelines to report this research ^4^.

## Results

Of 194 patients transferred to BRH from 28 April 2020 to 7 September 2020, 41 (21%) were admitted for continued recovery from confirmed COVID-19 pneumonia. Selected demographics and patient characteristics are shown in Table 1. The length of stay (LOS) at the transferring acute care hospital was median 42 [8–78] days for the post-COVID pneumonia cohort, with median 38 [8–77] days spent in the ICU. To contrast, the LOS for non-COVID patients admitted during the same time period was median 16 [1–96] days with median 15 [0-89] days spent in the ICU. All mechanical ventilation and hemodialysis interventions were initiated at the transferring hospital. Table 2 presents treatment interventions already in effect on admission to BRH, descriptive characteristics, and laboratory values. None of the seven patients excluded from weaning were chronically ventilated prior to admission to the transferring hospital. Upon evaluation by the consulting pulmonologist on admission to our LTAC, patients were determined not to be weaning candidates for the following reasons: physiologic instability (unmet readiness to wean parameters), and poor mentation or neurocognitive disorders.

**Table 1.** Selected demographics and characteristics of patients admitted for post-COVID recovery.

Variable	n=41
Age, years (median [range])	68 [44–94]
Gender, male (%)	61
Premorbid location, home (%)	68.3
Race/ethnicity (n (%))	
* African American*	2 (4.9)
* Asian/Pacific Islander*	6 (14.6)
* Caucasian*	15 (36.6)
* Hispanic*	18 (43.9)
COVID-19 risk factors (n (%))	
* Type 2 diabetes mellitus*	26 (63.4)
* Hypertension*	31 (75.6)
* Coronary artery disease*	11 (26.8)
* Hyperlipidemia*	15 (36.6)
*Obesity (BMI ≥ 30)*	16 (39)
At transferring hospital (n (%))	
* ARDS*	16 (39)
* Sepsis/septic shock*	20 (48.8)
* Invasive mechanical ventilation*	36 (87.8)
* Tracheotomy*	33 (80.5)
* Acute kidney injury/acute renal insufficiency*	21 (51.2)
* Heart failure*	12 (29.3)
Transferring hospital ICU/CCU days (median [range])	38 [8–77]
Transferring hospital length of stay, days (median [range])	42 [8–78]

**Table 2.** Status of post-COVID recovery patients on admission (n=41).

Variable	n(%)
Invasive mechanical ventilation	21 (51.2)
* Admitted for weaning*	14 (67)
Tracheostomy tube	33 (80.5)
Hemodialysis	9 (22)
Enteral feeding tube	32 (78)
Central line	20 (48.8)
Indwelling urinary catheter	17 (41.5)
Pressure injury ≥ stage 2	32 (78)
Multiple pressure injuries	19 (46.3)
Laboratory values (mean (SD)) **	
Serum albumin (g/dl)	2.82 (0.61)
Hematocrit (%)	30.0 (5.8)
BUN (mg/dl)	40.1 (26.3)
Creatinine (mg/dl)	1.43 (1.85)
Glucose (mg/dl)	146.6 (51.9)

## Discussion and conclusions

LTAC hospitals provide specialized care for patients suffering from CCI ^5^. With increased survival in the ICU, the number of patients transferred to these hospitals has also increased in the past decades ^2^. Early reports of the COVID-19 pandemic indicate that 5–12% of patients with COVID-19 infection require ICU hospitalization ^6– 8^. These numbers suggest that the role of LTAC hospitals will expand during the COVID-19 pandemic, due in part to their ability to treat patients with illnesses and conditions that do not follow a linear trajectory of improvement.

To our knowledge, this is the first report to characterize CCI and medically complex COVID-19 patients transferred to the post-acute venue of an LTAC hospital. Patients on average spent over six weeks in the transferring hospital mostly in the ICU, are largely elderly, carry the known risk factors for COVID-19 infection, and experienced respiratory failure necessitating prolonged mechanical ventilation via tracheostomy. Patients presented with physiological imbalances, numerous penetrating and indwelling catheters and disruptions of skin integrity breaching host defenses, and manifestations of allostatic load burden. Although our data reflect that post-COVID patients spent considerably more time at the transferring hospital than their non-COVID counterparts, notably in the ICU, we have included these early numbers simply as informational to satisfy any curiosities.

Our central purpose in reporting these data at this relatively early stage of the COVID-19 pandemic in southern California was to quickly share with critical care providers the characterization of the population of post-COVID infection patients admitted to our facility. While acute care hospitalizations were rapidly rising for COVID-19 illness, transfers to BRH continued to represent a general mix of patients with a variety of etiologies of CCI, among which 21% were admitted for treatment of post-COVID pneumonia.

Overall, our findings suggest that these patients will continue to require considerable medical interventions and treatments, including weaning from mechanical ventilation, owing to the numerous sequelae of the infection and the burden of acute-on-chronic diseases. As ICU survival rates improve, this research further emphasizes the important role of the LTAC in responding to the COVID-19 crisis. LTAC hospitals will play an increasingly critical function to fill gaps in our preparedness and response to COVID-19 infection by resuming and relieving care initiated in the acute hospital setting.

Our analysis is limited by several factors: it is a single center descriptive report, with a small cohort of patients, and a still emerging evidence base for COVID and post-COVID infection. Patient characteristics from this single center study may not be applicable to other centers or the post-COVID pneumonia population in general due to geographic differences in patient demographics, referral patterns, and facility-specific treatment capabilities. Efforts to quantify disease burden and report the number and variety of interventions may be warranted to objectify the intensity of treatment at the LTAC hospital. We look forward to reporting broad clinical outcomes (wean rate, time to wean, length of stay, functional status, and discharge disposition) from post-COVID patients admitted to BRH, with selected comparisons to the non-COVID patient population.

## Data availability

Open Science Framework: Database, https://doi.org/10.17605/OSF.IO/VHJZG ^9^. Registered 8 ^th^ October 2020 ( https://osf.io/2c8q9).

Data are available under the terms of the Creative Commons Zero "No rights reserved" data waiver (CC0 1.0 Public domain dedication). 
