# Supplementary material for: Post-COVID recovery: characteristics of chronically critically ill patients admitted to a long-term acute care hospital
Source: F1000Res. 2021 Apr 20;9:1241. Originally published 2020 Oct 15. [Version 2] doi: 10.12688/f1000research.26989.2 (PMC8108550; doi:10.12688/f1000research.26989.2)
Supplement: Peer review history

# Peer review: Reviewer response for version 2

**DOI:** 10.5256/f1000research.56115.r83595

**Published:** 2021-05-05

**Authors:** Anuj Mehta

**Affiliations:** 1 Division of Pulmonary, Critical Care, and Sleep Medicine, Department of Medicine, Denver Health and Hospital Authority, Denver, CO, USA

The authors have addressed all of my concerns including the primary purpose of the manuscript not being focused on outcomes. I have no further comments.

Is the work clearly and accurately presented and does it cite the current literature?

Yes

If applicable, is the statistical analysis and its interpretation appropriate?

Yes

Are all the source data underlying the results available to ensure full reproducibility?

No

Is the study design appropriate and is the work technically sound?

Yes

Are the conclusions drawn adequately supported by the results?

Yes

Are sufficient details of methods and analysis provided to allow replication by others?

Yes

Reviewer Expertise:

Critical care outcomes, shared decision-making, prolonged mechanical ventilation.

I confirm that I have read this submission and believe that I have an appropriate level of expertise to confirm that it is of an acceptable scientific standard.

# Peer review: Reviewer response for version 1

**DOI:** 10.5256/f1000research.29812.r77537

**Published:** 2021-02-15

**Authors:** Anil Makam

**Affiliations:** 1 Division of Hospital Medicine, San Francisco General Hospital and Trauma Center, Department of Medicine, University of California, San Francisco, San Francisco, CA, USA

The authors conducted a straightforward small descriptive study of COVID-19 patients, mostly who had CCI, who were transferred to a single non-profit LTACH in Southern California. The report describes baseline sociodemographic details and clinical characteristics at the preceding transferring hospital and upon admission. This small descriptive cohort study provides novel data that a small but meaningful population of COVID-19 patients experience CCI from COVID-19, and implies (but does not show) that they likely will suffer from post-ICU syndrome even after their LTACH stay. I have a few comments:

1. As the pandemic evolved, hospitals and clinicians gained experience in caring for this population. Were patients transferred later during the study period different that patients transferred earlier on?
2. Please provide the median LOS for non-COVID patients to support the claim that the preceding hospital LOS for COVID-19 patients was twice that of non-COVID-19 patients. Also, the interpretation of this is unclear, as COVID-19 patients may not have been transferred due to unfamiliarity with the disease course and isolation requirements, and not necessarily because of differences in severity/complexity of illness
3. What data were missing - just albumin and glucose? The sentence is awkwardly written, please correct.
4. Why were only 14 of 21 patients on invasive MV admitted for weaning? Were the other 7 chronically ventilated? 

Is the work clearly and accurately presented and does it cite the current literature?

Yes

If applicable, is the statistical analysis and its interpretation appropriate?

Yes

Are all the source data underlying the results available to ensure full reproducibility?

No

Is the study design appropriate and is the work technically sound?

Yes

Are the conclusions drawn adequately supported by the results?

Yes

Are sufficient details of methods and analysis provided to allow replication by others?

Yes

Reviewer Expertise:

LTACHs, health services research, comparative effectiveness, geriatrics, post-acute care, policy.

I confirm that I have read this submission and believe that I have an appropriate level of expertise to confirm that it is of an acceptable scientific standard.

# Response

**Published:** 2021-04-12

**Authors:** Meg Hassenpflug

**Affiliations:** Barlow Respiratory Hospital, USA

We kindly thank the Reviewer for his thoughtful comments and suggestions for our paper. Our responses follow:

  

1. The Reviewer notes that as the pandemic evolved, hospitals and clinicians gained experience in caring for this population. Were patients transferred later during the study period different that patients transferred earlier on? Referring to the study inclusion dates, our paper reflects admissions during roughly a four month time period. In view of the brief snapshot in time, along with a small cohort of patients we did not attempt to compare time periods at this point. We agree that such a comparison would be of interest and we will explore three, four, and six month time intervals as we build on this effort.
2. An oversight on our part in the Abstract and Results is that we failed to include the actual data on length of stay (LOS) at the transferring acute care hospital. We regret the omission and have excluded the related statement from the Abstract, and added the data to Results in the revised text. The LOS at the transferring acute care hospital was median 42 [8-78] days for the post-COVID pneumonia cohort, and median 16 [1-96] days for non-COVID patients admitted during the same time period. We made no attempt to interpret the data, and considered this simply an interesting informational contrast to be made at that point in time.
3. Regarding missing data: The Reviewer notes that the statements regarding missing data and the various “n” for the laboratory values serum albumin and serum glucose were perhaps awkwardly written. We were informing the reader that these two variables presented in Table 2 were not reflective of the entire cohort of 41 patients due to a few missing values. The text has been revised.
4. The Reviewer observes that just 14 of the 21 patients on invasive mechanical ventilation were admitted for weaning. He poses the question on whether the other seven patients were chronically ventilated. We welcome the question and thank the Reviewer for his observation. None of the seven patients excluded from weaning were chronically ventilated prior to admission to the transferring hospital. Upon evaluation by the consulting pulmonologist on admission to our LTAC, patients were determined not to be weaning candidates for the following reasons: physiologic instability (unmet readiness to wean parameters), poor mentation or neurocognitive disorders. We have now included this information in the revised text. Thank you so much for the question.

# Peer review: Reviewer response for version 1

**DOI:** 10.5256/f1000research.29812.r77597

**Published:** 2021-02-08

**Authors:** Anuj Mehta

**Affiliations:** 1 Division of Pulmonary, Critical Care, and Sleep Medicine, Department of Medicine, Denver Health and Hospital Authority, Denver, CO, USA

Thank you for allowing me the opportunity to review “Post-COVID recovery: characteristics of chronically critically ill patients admitted to a long-term acute care hospital” for consideration for your journal. The authors present an early case series of patients in the post-COVID-19 state who were admitted to an LTAC. They describe a significant burden of disease that highlights the need for ongoing care after initial recovery from acute COVID-19 illness. The authors present the experience of Barlow Respiratory Hospital which is a unique and large LTAC with a large referral base. The primary strength of the manuscript is that it presents novel data and gives a picture of the ongoing burden of disease related to COVID-19. However, the two major gaps are a sense of outcomes from the LTACH (which may be difficult to quantify as some patients may still be receiving treatment) and a sense of context with comparisons to the prior year. I have listed my specific concerns below. The source data is not included but is presented in an appropriate manner. 

**Major Comments**

1. The question that naturally stems from the authors description is what happened to these patients. It would be helpful for the authors to present some outcomes data including percent successfully weaned, percent with ongoing ventilation, percent died, percent transferred back to a short term acute care hospital, percent discharged home etc. It would also be helpful to understand patients’ functional status if the data is available. Such descriptions would be helpful to truly quantify not only the burden of disease but the likelihood of recovery.
2. The authors’ data indicates that 51.2% of post COVID-19 patients required MV and 22% required RRT of some form. It is well known that during the first spike in COVID in the spring, there was a substantial drop in other disease states presenting to the hospital. In essence, for a while most hospitals were seeing COVID and nothing but COVID. I am unclear as to how much this was true in Southern California but it is a phenomenon seen in multiple parts of the country. Therefore, comparing the COVID patients to the non-COVID patients during the same time may not be the best comparison. It would be interesting, if possible, to compare the COVID-19 patients admitted during the study period to patients admitted the year prior in terms of rates of MV, HD, tracheostomy, LOS at originating hospital, etc. This would provide broader context for what is truly COVID related.

**Minor Comments**

1. In the Abstract it is stated “Median age: 68 [44-94] years, 61% male, 80.5% with tracheostomy, 51.2% on invasive mechanical ventilation, 22% receiving hemodialysis”. It is confusing as to whether 80.5% of the 41 patients had a tracheostomy or 80.5% of patients who were receiving MV had a tracheostomy. Please clarify.
2. In the Methods it states “Missing data were omitted from analysis. For the variables, serum albumin and serum glucose, n=36 and n=40 respectively.” I am not sure what is meant by these sentences. Please clarify.

Is the work clearly and accurately presented and does it cite the current literature?

Yes

If applicable, is the statistical analysis and its interpretation appropriate?

Yes

Are all the source data underlying the results available to ensure full reproducibility?

No

Is the study design appropriate and is the work technically sound?

Yes

Are the conclusions drawn adequately supported by the results?

Yes

Are sufficient details of methods and analysis provided to allow replication by others?

Yes

Reviewer Expertise:

Critical care outcomes, shared decision-making, prolonged mechanical ventilation

I confirm that I have read this submission and believe that I have an appropriate level of expertise to confirm that it is of an acceptable scientific standard, however I have significant reservations, as outlined above.

# Response

**Published:** 2021-04-12

**Authors:** Meg Hassenpflug

**Affiliations:** Barlow Respiratory Hospital, USA

We kindly thank the Reviewer for his thoughtful comments and suggestions for our paper. Our responses follow:

 Major Comments

- We absolutely agree that what happens to these patients (outcomes) is of foremost interest. Referring to the study inclusion dates, this group of patients represents the beginning of the COVID pandemic in southern California. Our central purpose at this relatively early stage of the pandemic was to quickly share with critical care providers the characterization of the population of post-COVID infection patients admitted to our facility, to underscore the important role of the LTAC in responding to the COVID-19 crisis. It was not our intent to present outcomes at this early timepoint. With a small cohort of patients, and only 14 patients admitted for ventilator weaning, we believe that attempting to present any outcomes data may have been misleading.
- We agree that it would be helpful to understand patients’ functional status relative to a variety of specific outcomes (our interpretation of the phrase “likelihood of recovery”). These data were not included in the initial characterization of the population as it was not our intent to present any outcomes analysis at this early timepoint.
- The Reviewer’s point on the COVID vs non-COVID patient populations is well taken. The only minor comparison mentioned in the text between the two groups was length of stay (LOS) at the transferring hospital. An oversight on our part in the Abstract and Results is that we failed to include those data. We regret the omission and have excluded the related statement from the Abstract, and added the data to Results in the revised text. The LOS at the transferring acute care hospital was median 42 [8-78] days for the post-COVID pneumonia cohort, and median 16 [1-96] days for non-COVID patients admitted during the same time period. We made no attempt to interpret the data, and considered this simply an interesting informational contrast to be made at that point in time. We very much appreciate your suggestion to compare the COVID-19 patients admitted during the study period to patients admitted the year prior in terms of rates of MV, HD, tracheostomy, LOS at originating hospital, etc, and are doing so as we build on this effort. Thank you.

Minor Comments

1. In the Abstract, the Reviewer asks for clarification regarding whether 80.5% of the 41 patients had a tracheostomy or 80.5% of patients who were receiving MV had a tracheostomy. The percentages refer to the entire cohort, but we see where confusion could arise. The Abstract has been edited for clarity.
2. The Reviewer asks for clarification of statements regarding missing data and the various “n” for the laboratory values serum albumin and serum glucose. We were informing the reader that these two variables presented in Table 2 were not reflective of the entire cohort of 41 patients due to a few missing values. The text has been revised.
